# Complicated hepatic cyst with an incidental pelvic hydatid cyst: a case report

**DOI:** 10.1097/RC9.0000000000000973

**Published:** 2026-09-11

**Authors:** Nawwar Shaheen, Samer Sara

**Affiliations:** Faculty of Medicine, Damascus University, Damascus, Syrian Arab Republic

**Keywords:** cystic echinococcosis, extrahepatic, hydatid cyst, pelvic cyst, surgical management

## Abstract

**Introduction:**

Hydatid disease, a zoonosis caused by *Echinococcus granulosus*, most commonly affects the liver and lungs. Primary pelvic localization is exceptionally rare (< 2% of cases) and poses significant diagnostic challenges, particularly in patients without classic epidemiological risk factors. This case report highlights the complexities of managing such an atypical presentation.

**Case presentation:**

A 56-year-old Arab man from an urban area, with a history of mechanical aortic valve replacement and treatment with warfarin, presented with acute abdominal pain and fever. Imaging revealed a complicated, likely infected hepatic cyst in segment IV and an incidental presacral cystic lesion. Serology was positive for *E. granulosus*. Emergency laparotomy confirmed that both cysts were hydatid in origin. Management included warfarin reversal, surgical evacuation of both cysts with scolicidal irrigation, and post-operative albendazole therapy. The patient remained healthy, with a good recovery at the 6-month follow-up.

**Clinical discussion:**

The concurrent presence of a symptomatic hepatic cyst and an asymptomatic primary pelvic cyst is rare. This case underscores that hydatid disease can occur in urban patients without a typical exposure history, necessitating a high index of suspicion based on imaging. The acute presentation due to cyst superinfection required urgent surgical intervention. Successful management hinges on meticulous surgical technique to prevent spillage, combined with adjunctive anthelmintic therapy. Long-term surveillance is essential to monitor for recurrence.

**Conclusion:**

Hydatid disease should be considered in the differential diagnosis of cystic abdominal masses even in low-risk populations. A structured approach involving timely imaging, careful surgical management, peri-operative albendazole, and prolonged follow-up may be crucial for optimal outcomes in complex, multi-focal cases.

## Introduction

Hydatid disease, or cystic echinococcosis, is a persistent global zoonosis caused by the larval stage of the tapeworm *Echinococcus granulosus*^[^1–3^]^. While advances in imaging and treatment have improved outcomes, the disease remains a significant public health challenge, particularly due to its capacity for atypical and clinically deceptive presentations^[^3–6^]^.

The classic pathogenesis involves humans as accidental intermediate hosts. After ingestion, the embryos most commonly embolize in the liver (55–70% of cases) and lungs (18–35%), acting as the primary vascular filters^[^2,4^]^. Consequently, the vast majority of clinical attention focuses on hepatopulmonary involvement.

However, a small but significant minority of embryos bypass these filters, leading to primary cysts in virtually any anatomical site^[^3,5^]^. Among these extrahepatic locations, pelvic and retroperitoneal hydatid cysts are exceptionally rare, representing less than 2% of cases in endemic regions^[^4,7^]^. Their pathogenesis is theorized to involve hematogenous or lymphatic dissemination, or secondary spread from a ruptured abdominal cyst^[^3,5^]^.

HIGHLIGHTS

- Concurrent primary pelvic and complicated hepatic hydatid cysts represent an exceptionally rare clinical presentation.
- A high index of suspicion for cystic echinococcosis is essential, even in urban patients without classic epidemiological risk factors.
- Successful management requires a structured protocol: urgent surgery with meticulous scolicidal technique, combined with peri-operative albendazole and long-term surveillance.

These unusual localizations pose a formidable diagnostic challenge. Cysts grow slowly and may remain asymptomatic for years until they exert a mass effect on adjacent structures^[^2,7^]^. Clinical presentations are therefore nonspecific and location-dependent. A pelvic cyst may present with lower abdominal pain, urinary frequency, or acute urinary retention^[^6–8^]^. In the absence of classic epidemiological clues, the initial differential diagnosis rarely includes hydatidosis, often leading to delays and misdiagnosis as more common conditions such as benign tumors or abscesses^[^1,3,5^]^.

Imaging is the cornerstone of diagnosis. Ultrasound is the recommended first-line modality and can reveal pathognomonic signs^[^1,6^]^. For preoperative planning in complex pelvic or retroperitoneal locations, contrast-enhanced computed tomography (CT) or magnetic resonance imaging is invaluable^[^4,5^]^. Serological tests, while supportive, can yield false-negative results, particularly in isolated extrahepatic disease^[^1,3^]^.

The management of hydatid disease, especially in complex locations, is multimodal. Surgery remains the definitive treatment, with the goal being complete cyst excision without spillage, to prevent anaphylaxis and secondary seeding^[^2,4,6^]^. Meticulous surgical technique, including the use of scolicidal agents, is paramount^[^4,6^]^. Medical therapy with albendazole serves as a crucial adjunct to reduce the risk of recurrence^[^1,6^]^.

This case study presents a 56-year-old Arab man with an acute abdominal presentation who was ultimately found to have a concurrent complicated hepatic cyst and a primary pelvic hydatid cyst.

This case report adheres to the Revised Surgical CAse REport (SCARE) guidelines as outlined by Kerwan *et al*^[^9^]^.

## Case presentation

A 56-year-old Arab man from an urban center presented to the emergency department with acute abdominal pain and right upper quadrant discomfort for 48 hours. His past medical history was significant for a mechanical aortic valve replacement in 2018, for which he was on long-term warfarin therapy. He had no known history of hydatid disease, no history of animal contact, and no recent travel to endemic areas. Socially, he was a smoker with a 40-pack-year history and denied alcohol use. Family history was non-contributory. He described the sudden onset of severe, constant pain, stating, “It was a stabbing pain under my ribs that got worse every time I moved or took a deep breath.”

On admission, his vital signs were a temperature of 38.5°C, a blood pressure of 150/95 mmHg, a heart rate of 115 bpm, and a respiratory rate of 22 breaths/min. Physical examination of the abdomen revealed tenderness and guarding in the right hypochondrium and epigastrium, with a positive Murphy’s sign; no masses were palpable, and bowel sounds were hypoactive. The initial clinical assessment considered acute cholecystitis, a perforated peptic ulcer, and a complicated hepatic cyst in the differential diagnosis.

The timeline began with the onset of pain on Day 1. He presented to the Emergency Department on Day 3, where blood tests and an ultrasound were performed, leading to the decision for an emergency exploratory laparotomy that same day. The surgery involved the evacuation of a hepatic cyst in Segment IV and of an incidentally discovered pelvic cyst.

Pre-operative laboratory investigations revealed leukocytosis of 17 500/µL with neutrophil predominance; a hemoglobin level of 12.8 g/dL; and amylase and lipase within normal limits. Liver function tests were elevated, with an alanine aminotransferase of 85 U/L, an aspartate aminotransferase of 78 U/L, and an alkaline phosphatase of 210 U/L. Serology for *Echinococcus* was positive for *E. granulosus* (IgG) via ELISA. Due to his valve replacement, his coagulation profile showed an international normalized ratio of 3.8. Transabdominal ultrasound revealed a complex, mixed-echogenicity 6 × 4.6 cm cyst in liver segment IV-A with internal echoes suggestive of debris and gas, highly suspicious for a complicated, infected hydatid cyst, along with significant free fluid. A subsequent contrast-enhanced CT scan confirmed a thick-walled, partially calcified hepatic cyst in segment IV with internal septations and an air-fluid level, consistent with superinfection (Fig. 1), and also identified a well-defined, unilocular 5 cm cystic lesion in the presacral space. The pathologic diagnosis from intra-operative samples confirmed the presence of acellular laminated membranes and hooklets pathognomonic for *E. granulosus*.

**Figure 1.**
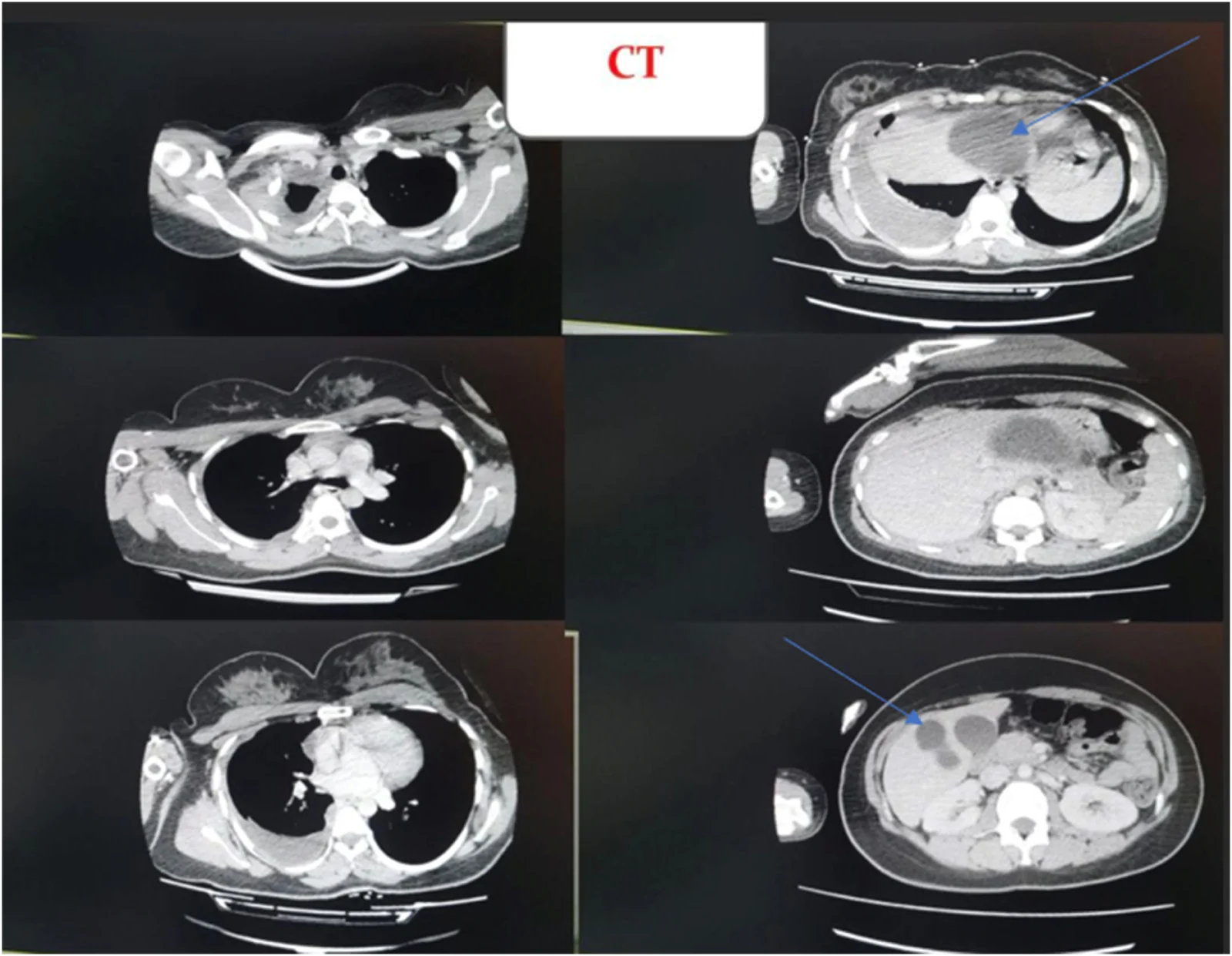
Contrast-enhanced CT scan of the abdomen and pelvis. (left) Axial view showing the hepatic cyst in segment IV with an air-fluid level (solid arrow) and internal septations. (right) Coronal view demonstrating the pelvic cyst anterior to the sacrum (dashed arrow).

The differential diagnosis for the pelvic lesion included an ovarian cyst or tumor (excluded by imaging showing the lesion anterior to the sacrum and separate from the adnexa, and by intraoperative confirmation), presacral teratoma (excluded by lack of fat or calcific densities on CT and the presence of pathognomonic hydatid membranes on pathology), pelvic abscess (excluded by the absence of systemic sepsis parameters at baseline and the clear, colorless fluid without purulence at surgery), and an epidermoid or dermoid cyst (excluded by imaging characteristics and histopathology).

Therapeutic intervention began pre-operatively with intravenous fluids, broad-spectrum antibiotics (piperacillin–tazobactam), analgesic management, and reversal of his warfarin with vitamin K and fresh frozen plasma. The surgical procedure via a midline exploratory laparotomy involved adhesiolysis and puncture of the hepatic cyst, which yielded turbid, brownish fluid mixed with pus and degenerated membrane material; the cavity was unroofed, irrigated copiously with hypertonic saline and then normal saline, followed by omentoplasty and placement of a closed suction drain (Figs. 2 and 3). The incidental pelvic cyst, identified anterior to the sacrum, was accessed through the same midline laparotomy, with the small bowel retracted superolaterally. The cyst was located medial to the right ureter (which was carefully identified and preserved), lateral to the rectum, and superior to the pelvic floor. The ureter was mobilized gently to provide safe access. The cyst was entered and evacuated of clear, colorless fluid containing multiple daughter vesicles. Unlike the hepatic cavity, the pelvic cavity was not amenable to omentoplasty; instead, it was irrigated copiously with hypertonic saline, followed by normal saline, and a small closed suction drain was placed to allow the cavity to collapse and heal by secondary intention. Post-operative medical therapy consisted of oral albendazole at 400 mg twice daily, initiated on post-operative Day 2 for a planned 6-month course.

**Figure 2.**
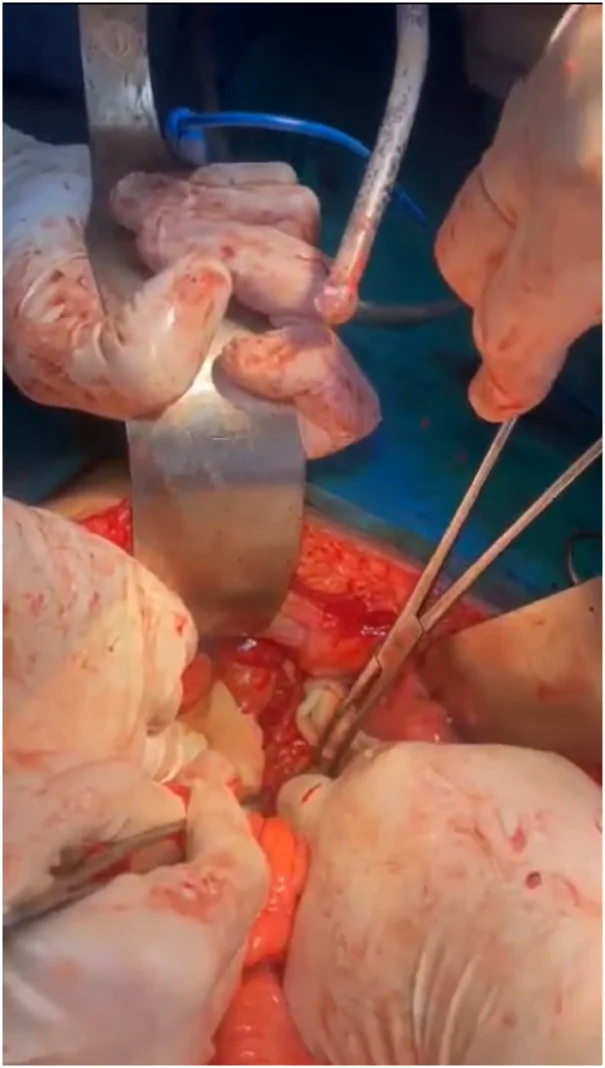
Intraoperative photograph showing the hepatic cyst cavity after unroofing. The arrow indicates the edge of the liver parenchyma; the asterisk marks the gallbladder for orientation.

**Figure 3.**
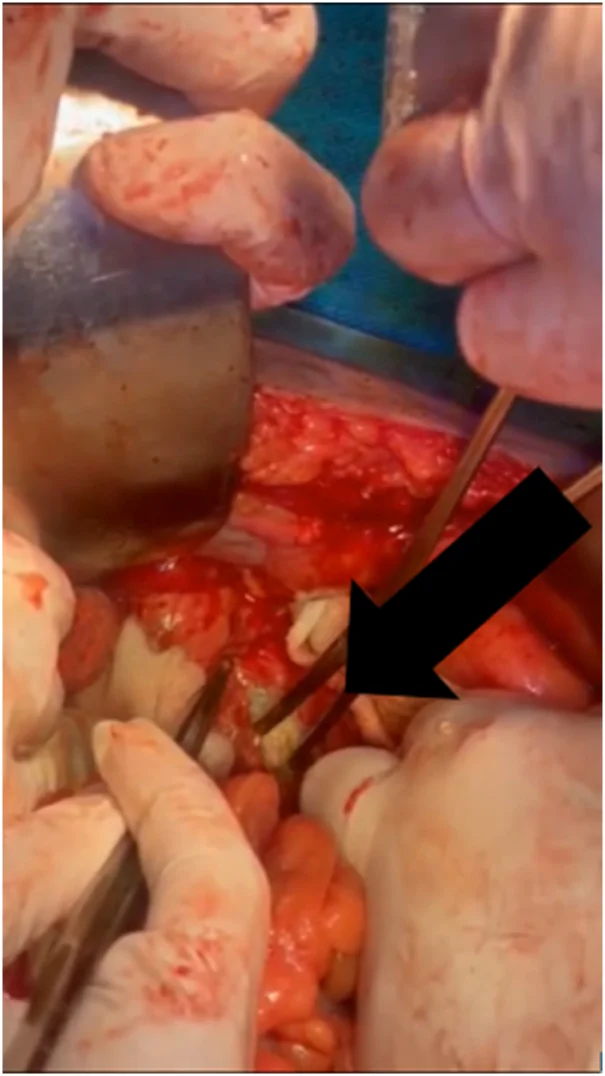
Intraoperative photograph of the pelvic cyst cavity. The arrow indicates the right ureter, which was carefully identified and preserved, and the sacral promontory is visible at the base of the field.

His in-hospital course was uneventful; the abdominal drain was removed on post-operative Day 4, and he was discharged on Day 7 in stable condition. At the 6-month outpatient follow-up, he was asymptomatic, and an ultrasound showed a significant reduction in the size of the liver cavity with no residual pelvic collection. Serological titers were scheduled for monitoring at 3, 6, and 12 months. Planned long-term monitoring includes annual ultrasound imaging for at least 5 years to monitor for recurrence.

## Discussion

This case of a 56-year-old urban Arab man with a concurrent complicated hepatic hydatid cyst and an incidental primary pelvic cyst offers valuable insights into the diagnostic and therapeutic complexities of cystic echinococcosis, particularly in non-classical presentations. It underscores several critical lessons pertinent to modern clinical practice.

The simultaneous presence of hepatic and primary pelvic hydatid cysts is exceptionally uncommon^[^8,10,11^]^. While the liver is the most frequently involved organ, primary pelvic hydatid disease is a rare entity, accounting for less than 2% of all intra-abdominal cases^[^12^]^. Pelvic cysts are thought to arise from hematogenous or lymphatic dissemination of oncospheres that bypass the hepatic and pulmonary filters, or less commonly, from secondary spread following rupture of an abdominal cyst^[^10,11^]^. The discovery of a pelvic cyst during surgery for a hepatic cyst, as in our patient, highlights the imperative for thorough intra-abdominal exploration in all hydatid disease surgeries. Similar cases of multi-focal abdominal disease have been reported, emphasizing the parasite’s potential for widespread dissemination^[^12,13^]^.

The importance of recognizing atypical extrahepatic localizations cannot be overstated. Isolated cases of hydatid disease in unexpected anatomical sites have been documented, including spinal hydatid presenting as lumbar radiculopathy and cerebellar hydatid in pediatric patients^[^14,15^]^. These reports reinforce the concept that oncospheres can seed virtually any anatomical compartment, making a high index of suspicion crucial, regardless of location or patient history.

Our patient’s profile – an urban dweller with no history of animal contact or travel to endemic regions – initially placed hydatid disease low on the differential. This scenario mirrors the growing recognition of “autochthonous” or sporadic cases in non-endemic urban settings, which pose a significant diagnostic dilemma^[^8,11^]^. In such contexts, the lack of a classic epidemiological history can lead to missed or delayed diagnosis, as clinicians may not consider echinococcosis^[^12,13^]^. Our case reinforces that a high index of suspicion must be maintained based on imaging findings, regardless of patient-reported risk factors. The diagnostic journey often begins with ultrasound, but as demonstrated, CT scanning is crucial for characterizing complex cysts, planning surgery, and detecting unsuspected secondary sites.

The patient’s acute presentation with signs of infection (fever, leukocytosis) and an air-fluid level on imaging indicated a superinfected hepatic cyst, a serious complication. This shift from an indolent parasitic cyst to an acute surgical abdomen is a well-documented challenge^[^16^]^. The presence of such complications often narrows the therapeutic window, necessitating urgent intervention to address sepsis and prevent catastrophic rupture^[^17^]^. This contrasts with the more common asymptomatic or incidentally discovered cysts, which allow for elective planning.

The management of this case involved a nuanced, multi-step approach:

Preoperative stabilization: Reversal of warfarin anticoagulation was a critical first step, underscoring the need to tailor emergency surgical planning to an individual patient’s comorbidities.

Surgical strategy: The midline laparotomy allowed for comprehensive exploration and simultaneous management of both cysts. The principles applied – careful puncture, aspiration, copious irrigation with a scolicidal agent (hypertonic saline), and omentoplasty for the liver cavity – constitute the established standard to minimize spillage and recurrence^[^12,16^]^. For the pelvic cyst, access required meticulous dissection to identify and preserve the ureter, with the residual cavity managed by irrigation and drainage rather than omentoplasty due to anatomical constraints.

Scolicidal irrigation and systemic implications: Hypertonic saline (30%) was used as the scolicidal agent of choice. While effective, its systemic absorption can lead to significant electrolyte disturbances, including hypernatremia. Prospective data have demonstrated clinically meaningful electrolyte shifts following irrigation with hypertonic saline during hydatid cyst surgery^[^18^]^. This is particularly relevant in patients with complex perioperative profiles, such as our patient, who was already anticoagulated and had systemic infection. Careful irrigation technique, limited dwell time, and copious subsequent irrigation with normal saline are essential to mitigate these risks, along with perioperative monitoring of serum sodium levels.

Adjunct medical therapy: The postoperative initiation of albendazole is a cornerstone of modern management. This aligns with consensus guidelines that recommend perioperative chemotherapy to reduce the risk of recurrence from microscopic residual disease^[^8,16^]^. The planned 6-month course is consistent with common protocols for disseminated or complex disease.

Long-term follow-up is paramount in hydatid disease. Our plan for serial ultrasound and serology (IgG titers) aligns with standard monitoring protocols to detect early recurrence^[^12^]^. Patients with disseminated or extrahepatic disease may require extended surveillance beyond the typical 3–5 years. The patient’s asymptomatic status and a reduced cyst cavity at 6 months are favorable early indicators, but sustained follow-up is necessary to assess for definitive cure.

## Conclusion

In summary, this case illustrates that hydatid disease can present acutely in urban patients without classic risk factors, often with complex manifestations like concurrent multi-organ involvement. It reinforces that the diagnosis hinges on imaging acumen, and successful outcomes depend on a structured approach: timely surgical intervention using meticulous techniques to prevent spillage, combined with pre- and post-operative albendazole therapy, while remaining vigilant for the systemic effects of scolicidal irrigation. Finally, it underscores the necessity of long-term surveillance to maximize the chances of a lasting cure. For clinicians worldwide, this case serves as a reminder to consider echinococcosis in the differential diagnosis of cystic abdominal masses, even in seemingly low-risk populations.

## Data Availability

Not applicable.
